# A Case Report of Schizoaffective Disorder with Pseudoseizures in a 42-year-old Male

**DOI:** 10.7759/cureus.4835

**Published:** 2019-06-05

**Authors:** Emmanuel Tito, Blayne Knapp, Anthony Bucca, Eduardo D Espiridion

**Affiliations:** 1 Internal Medicine, West Virginia School of Osteopathic Medicine, Lewisburg, USA; 2 Family Medicine, West Virginia School of Osteopathic Medicine, Lewisburg, USA; 3 Miscellaneous, West Virginia School of Osteopathic Medicine, Lewisburg, USA; 4 Psychiatry, Frederick Memorial Hospital, Frederick, USA

**Keywords:** schizoaffective disorder, schizophrenia, seizures, psychogenic non-epileptic seizures, epilepsy, conversion disorder, psychosocial, psychiatric, electroencephalogram

## Abstract

Psychogenic nonepileptic seizures (PNES), historically referred to as pseudoseizures or hysterical seizures, are sudden disturbances of motor, sensory, autonomic, cognitive, or emotional functions that can mimic epileptic seizures. PNES have a psychologic etiology related to dissociative disorders or conversion disorders, as opposed to the abnormally excessive neuronal activity found in epileptic seizures. Psychosocial conflicts are essentially converted into physical symptoms, resulting in seizure-like symptoms. This case report presents a 42-year-old male with a past history of child abuse, drug abuse, schizoaffective disorder, prior psychiatric hospitalizations, and diabetes mellitus type 2 who was admitted to the behavioral health unit with recurrent seizure-like episodes. These episodes were witnessed in the ED, however, appropriate workup failed to confirm abnormal neural activity or evidence of any brain injury. The patient was admitted to the psychiatric service where he was monitored for additional epileptic activity using long-term video-electroencephalogram (EEG) monitoring (LT-VEEG). While subsequent clinical events that resembled epileptic seizures were observed, the LT-VEEG failed to identify any epileptic activity. A diagnosis of PNES was established and a decision was made for the patient to remain on his current antiepileptic, psychiatric, and diabetes medications during his hospitalization. He showed gradual improvement during his stay and confirmed an understanding of his diagnosis. He was released three days later with instructions for follow-up and continued treatment with his outpatient psychiatrist. Early therapeutic counseling in patients who have a differential diagnosis of PNES should be considered to help identify and address the underlying causes of the seizure activity in an effort to curtail these seizure-like events.

## Introduction

Witnessed episodic neurologic events are frequently a challenging presentation for providers. This is especially true when they are associated with changes in mentation and significant motor activity. To the lay public, such an episode would appear to be of epileptic origin, commonly known as a seizure. To the clinician, however, it is essential to approach this presentation with a broad differential diagnosis in order to prevent mismanagement. An important item to have on the list of differentials is a disorder of psychopathologic origin known as psychogenic nonepileptic seizures, or PNES. PNES is not caused by abnormal brain electrical activity. PNES is characterized as episodic disturbances in cortical function that mimic epilepsy but are not associated with excessive neuronal activity and instead are the result of psychologic processes [1-2].

Psychogenic nonepileptic seizures are generally understood to be associated with a history of physical or sexual abuse, developmental stressors, post-traumatic stress disorder (PTSD), and dissociative or conversion disorders, though characterizing PNES continues to represent a challenge for medical practitioners. In part, these challenges are due to the heterogeneity of patients who suffer from PNES, the presentation of the disorder, and the way providers have historically defined the disorder as a medical community. Diagnostic criteria for PNES were revised in the Diagnostic and Statistical Manual of Mental Disorders, Fifth Edition (DSM-5) to rule in a diagnosis of conversion disorder, whereas in the past, PNES was a diagnosis separate from conversion disorder [3-4]. PNES has also been described in association with dissociative disorders, borderline personality disorders, and neuroticism [5]. From a psychodynamic perspective, PNES generally functions as a coping mechanism in patients that have suffered from psychiatric illness or past history of abuse. Patients tend to have a history of maladaptive coping strategies, however, PNES is presumed to be separate from malingering and factitious disorders in that episodes are not intentional or of conscious, controllable origin [1, 6].

In terms of defining guidelines to assess for PNES, further work must still be done to allow clinical differentiation of PNES presentation from epilepsy. The increasing availability of the electroencephalogram (EEG) with video functions has proven to be current gold standard in its ability to monitor neuronal activity and provide objective discrimination of PNES from epilepsy, but EEG is expensive, time consuming, and not readily available across all points of care [1, 7]. From the point of view of associated psychiatric comorbidities, it is important to gather a detailed history and physical assessment and screen the patient for any reports of abuse, psychiatric illness, history of trauma, or signs of other maladaptive coping mechanisms [4, 8]. Currently, what is known about factors that distinguish PNES from epilepsy revolve around the ability of the provider to keep a broad perspective of differential diagnoses and to gather a comprehensive history and review of systems from the patient. The importance of the practitioner to keep an open mind and not compromise the physician-patient relationship in a way that makes the patient feel their story of seizure-like symptoms is being dismissed has also been documented to be important in patient acceptance of appropriate medical treatment regimens.

Evidence-based recommendations for treatment for PNES are available, however, treatment guidelines are still lacking. There is evidence that discontinuation of any previously prescribed antiepileptic medication is encouraged as there is no benefit, it prolongs appropriate management of the disorder, contributes to healthcare and patient cost burdens, and exposes the patient to potential side effects of excess medications [1, 7, 9]. There are some studies that have shown decreased frequency of PNES events with nonpharmacologic management of reducing precipitating or perpetuating environmental triggers, behavioral modification therapies and cognitive behavioral therapy (CBT) techniques aimed at addressing any psychiatric co-morbidities, as well as guided transcranial direct current stimulation [9-12].

Further work is being done to characterize the psychopathologic origin of PNES and its association with psychosocial stressors, physical associations, risk factors, prevalence, and effective treatment modalities for the condition. Presented in this case report is a 42-year-old patient who was previously diagnosed with generalized seizures. The aim of this case report is to demonstrate the detail of patient history gathered on evaluation, clinical phenomena, and neurologic testing that were utilized to distinguish the diagnosis of PNES from epilepsy and contribute to literature in establishing more information on this disorder.

## Case presentation

A 42-year-old married Caucasian male with a history of schizoaffective disorder and prior psychiatric hospitalizations was admitted to the hospital ED for recurrent seizure episodes. The episodes occurred at home and were witnessed by the patient’s wife which prompted him to seek medical care. While in the ED, three episodes of plausible grand mal tonic-clonic seizures with loss of consciousness accompanied by urinary and bowel incontinence were witnessed. Episodes were followed by a postictal presentation which consisted of diaphoresis and confusion. No drowsiness or lethargy was reported. During the patient’s ED visit, he admitted to disturbing command auditory hallucinations that had been present for three months. The patient stated these hallucinations occurred when he was upset and that they involved two male voices commanding him to harm himself. These reports prompted his admission to the behavioral health unit for evaluation. When asked about possible causes of the seizure-like activity, the patient indicated that he had been without access to his medications for two weeks after missing a doctor’s appointment while travelling out of state to visit one of his daughters. The patient’s regular regimen included valproic acid 1000 mg twice daily and clonazepam 0.5 mg four times daily for seizures, fluoxetine 10 mg daily for depression, quetiapine 100 mg twice daily in addition to a 400 mg dose of quetiapine at bedtime for his schizoaffective disorder. The patient also reported regularly taking 30 units of insulin detemir twice daily for blood glucose control.

The patient’s past medical history was significant for longstanding psychiatric history with prior psychiatric hospitalizations following a suicide attempt, a diagnosis of schizoaffective disorder established at age 15, and recurrent seizures for the past four years. The patient reported that the seizures started at the same time that his grandmother had passed away. He also admitted to beginning illicit drug use including cocaine and opioids at that time, though he denied recent illicit substance use. Other medical illness history included depression, type 1 diabetes, blindness secondary to diabetic retinopathy, and psychiatric trauma of abuse sustained during his childhood. His family history was significant for mental illness, alcohol use disorder, child abuse, and suicide of a sibling.

On exam, the patient’s vital signs were within normal limits with the exception of an elevated blood pressure recording and hyperglycemia on initial physical exam. His complete blood count was within normal limits. His musculoskeletal exam was significant for gait ataxia and there was no evidence of decreased muscle strength. Given the witnessed seizure-like episodes in the ED, neurology was consulted to evaluate the patient.

In addition to neurology evaluation, a noncontrast head CT scan was ordered, and a preliminary EEG was performed. Neurologists’ in depth assessment and neurologic testing revealed unremarkable findings. The noncontrast CT scan imaging of the patient’s head was similarly unremarkable for any acute or subacute intracranial pathologies. The preliminary results did not show any epileptic focus recorded, which subsequently prompted long-term video-EEG monitoring (LT-VEEG) during the patient’s stay in the behavioral health unit.

During the LT-VEEG monitoring multiple clinical events were recorded on video. The patient was seen to have sudden onset of rapid and rhythmic head nodding, jaw jerking, subtle flexion of torso and occasional jerking of the left arm. However, there was no associated evidence of epilepticus focus on EEG other than muscle and motion artifacts. No focal abnormalities and no interictal epileptiform discharges were recorded. The continuous LT-VEEG results were normal in the patient’s waking, drowsy, and sleep states. The interpretation of the LT-VEEG findings revealed clinically observed events recorded on video with no associated EEG changes. Therefore, the events were concluded to be nonepileptic and psychogenic in nature. 

The patient’s psychiatric evaluation in the behavioral health unit revealed no psychomotor agitation or retardation, and no evidence of aggressive or agitated behaviors. His speech was coherent, organized, and goal-directed during the interview. He exhibited fair insight and judgment with adequate impulse control. He denied thoughts of broadcasting, insertions, or withdrawal. His mood was sad and affect was constricted, though he denied current suicidal or homicidal ideations, intents, or plans. There were referential and paranoid ideations present. The patient did admit to experiencing command auditory hallucinations. He denied problems with concentration, deficits in immediate retention or recall, and recent or remote memory on evaluation.

On completion of the interview, the patient agreed to admission to the behavioral health unit for observation, and the decision was made to continue all of his current medications. The patient showed gradual improvement over the next three days before discharge. He was advised to follow-up with his psychiatrist with plans to begin psychotherapy, as well as his endocrinologist to follow-up on diabetic blood glucose control. Psychoeducation was provided regarding medications and recommendations. The patient exhibited understanding of the EEG results, and displayed cooperation and motivation about the nature and extent of his condition, as well as the need for follow-up and continued treatment from his outpatient psychiatrist.

## Discussion

Psychogenic nonepileptic seizures are often misdiagnosed as epilepsy. While the exact pathology of PNES is not well understood, it can be a complex disorder to evaluate. A significant correlation between psychiatric illness and PNES has been documented but there is still no clear mechanism to explain the causality. Many studies have found an association between PNES and psychologic trauma such as physical and sexual abuse, personality disorder, affective disorder, or a history of PTSD [13-14]. The patient’s medical history was significant for schizoaffective disorder and child abuse. He also presented to the behavior health unit with hallucinations and suicidal thoughts. PNES can fall under the diagnosis of conversion disorder, factitious disorder, or malingering in the DSM-5. According to the DSM-5, PNES consists of involuntary movements that resemble epileptic seizures but lacks organic cause [3]. Psychologic stressors such as marital problems, death of a loved one, or health problems can trigger PNES. Patients with PNES are usually not able to suppress or control them. They often have a history of substance use disorder [15]. In this case, the patient reported using various illicit substances including cocaine and opioids following the death of his grandmother. He reported his seizures began at this time as well. Furthermore, PNES can involve secondary gain such as increased attention and release from unpleasant responsibilities [15].

During the episode of PNES witnessed in the ED the patient was unaware of his surroundings with limited consciousness and function after the event. In addition to repeated attacks of involuntary movements affecting all four extremities, he also suffered tongue biting and had loss in bladder control. These findings are rare in PNES. The seizure episodes lasted 30 s to 2 min. There were no associated epileptiform discharges recorded from scalp electrodes other than muscle and motion artifacts. An elevation of serum prolactin two times baseline can be taken as a predictor of a true epilepsy [16]. Though prolactin levels were not measured in this patient, it is often used to differentiate PNES from true epileptic seizures. It is possible that drug use and withdrawal can trigger seizures. However, his normal EEG reading suggests it was unlikely that he developed drug induced seizures. It can be difficult for both patients and healthcare professionals to discuss and treat PNES. Shen et al. proposed a method for explaining the diagnosis [17]. This approach appears to build from a physician-patient relationship to guide the treatment. It consists of explaining that the seizures are not epileptic in nature and there is no evidence of brain injury. The method also indicates that physicians should recommend psychotherapy and education on adopting healthy coping mechanisms to deal with ongoing stressors [17]. Finally, it suggests that physicians cease prescribing antiepileptic drugs, thus relieving the patients of associated side effects [1, 17]. Discontinuation of the patients’ antiepileptic medications upon discharge was acutely deferred as behavioral health practitioners restarted his full medication regimen during hospitalization and noted an improvement in his condition, despite lack of evidence of epileptic origin as explored with LT-VEEG. The reason for not discontinuing his antiepileptic medication regimen was due to the patient’s improvement in symptoms once his medications were restarted, after his reported lack of access to his medications for two weeks. Additional basis for this decision included the patient’s well-documented long-term relationship with his outpatient psychiatrist. Psychoeducation on the decision to defer discontinuation of the antiepileptic medication regimen was shared with the patient who expressed understanding to pursue this with his outpatient point of care on follow-up.

## Conclusions

Patients presenting with PNES ensue a lengthy diagnostic workup to rule out causes of epileptic activity. They face a challenging treatment plan that requires patient understanding of their condition, willingness to participate in psychotherapy to determine the underlying causes of their presentation, and cessation of their antiepileptic medications. Even with treatment, patients diagnosed with PNES have a low remission rate of seizure-like episodes. This underscores the importance of proper diagnosis by the physician, ensuring patient compliance with the treatment plan, routine therapy, and the need for additional studies to be completed to determine guidelines for assessing and treating PNES.
